# Miscellaneous Surgical Notes from Berlin

**Published:** 1879-04

**Authors:** W. S. Caldwell

**Affiliations:** London


### Foreign Correspondence.

### ARTICLE XI.

### MISCELLANEOUS SURGICAL NOTES FROM BERLIN.

As a place for the study of surgery, and the witnessing of surgical operations, Berlin is undoubtedly the very best point in all Europe, and hence probably the best in the world.

Other cities have as much or more surgical material, but it is generally, as here in London, so divided between different hospitals and different operators, that it is impossible for one to witness it all, or to be benefited by it, to such a degree as at "Langenbeck's Clinic" in the German capital. For from two to four hours each day, for six days in the week, you can witness here every variety of surgical operation, from the opening of an abcess to those the most grave known to the domain of surgery.

To-day Prof. Langenbeck is to surgery in Prussia, what no other man is to this branch of medicine in any other country.

Although he has introduced and is really the father of many of the great operations that he performs, he is a man without a hobby, and never speaks of any procedure as being an invention of his own. While Péan, Gosselin and Verneuil seldom quote anything but French authority, and Bryant, Lister and Paget are partial to British authors, Langenbeck is as cosmopolitan in the sources from which he draws his knowledge of the branch that he teaches, as the universe itself.

As a didactic teacher, he never would have been a success in America, where a certain eloquence of delivery is considered so essential in the lecture-room. In lecturing, he often hesitates, and will sometimes stop for several seconds between the phrases of his sentences. In treating of any surgical procedure of importance, he first gives the views and teachings of others, and then compares the same with his own experience; which, taking the whole field of surgery together, is probably the most varied and extensive of any man's now living. While Péan, of Paris, does only the most important part of his surgery, leaving the closing and dressing of wounds to his assistants, Langenbeck attends to every detail himself, ties every artery and puts in every suture.

With the use of the antiseptic spray, he says that *time* has ceased to be an essential factor in the performance of most surgical operations.

Hence he does his work in the most deliberate manner; without dash and without bustle, he executes his task with that steady and matter-of-fact composure with which a mechanic would use his tools in the pursuit of a trade to which he had devoted his entire life. Only in a single instance do I remember to have seen him show the least agitation, even in the greatest emergency.

A young man came to the "Clinic" who had a large fibroid growth situated in and attached to the superior and posterior aspect of the nares. Prof. Langenbeck first thought, on examination of the case, that it would be necessary to resect a portion of the superior maxillary bone in order to reach the mass and remove it. But finding, on further examination, that he could reach it by way of the pharynx, he laid the patient on his back on the operating table, and seizing the growth with a strong pair of forceps, with great force he twisted it off. So immense was the hæmorrhage that followed the operation, that the patient lost one liter of blood in less time than that in which the reader can read two lines across the pages of this journal. The old man stood amazed and cried out, "Good Lord, what shall I do? I think I shall have to ligate the carotids;" but with a presence of mind that did not even now desert him, he placed both thumbs on these vessels, and while he made firm pressure he had the man raised to the upright position, and by continuing the pressure the bleeding soon ceased. The doctor said that his first impression was, on seeing this great flow of blood, that the tumor was attached to the internal carotid, and that in tearing it away he had torn through the coats of this vessel.

The patient made a slow but perfect recovery.

Report says that this year will end the labors of this great man as a teacher, as he proposes at the end of the next Semester to resign his Professorship in the University of Berlin.

Prof. Volkmann of Halle, an eloquent teacher, and a man in the prime of life, is likely to take his place; while Dr. Schede, Surgical Director of the City Hospital of Berlin, and a man, by the way, who excels Lister himself in carrying out the principles of antiseptic surgery, will go as Professor of Surgery to Halle.

To select from the immense amount of material that I have had access to in this clinic, such as would be of most interest to the reader, is a task of no little perplexity; but I shall follow the same rule in my selections that has heretofore governed me, and that is, to give those cases the preference that are most likely to be met with and require management in ordinary practice.

### AMPUTATION OF THE FEMUR.

A man aged 36 years, suffering from a malignant disease of the lower end of the femur, was brought into the ampitheater, and while the assstants were administering chloroform to the patient, and making other necessary arrangements for the amputation, Prof. Langenbeck made the following general remarks on the subject:

"Where it can be done, I make *side* flaps, instead of antero-posterior ones, as is generally done. I do this, first, because a better drainage can be effected after the operation, and second, because I believe any resulting cicatrix is more likely to be situated at a point that will render it less liable to be irritated by the friction connected with the movements of the stump.

"Again, I make my flaps *entirely* of skin and its immediately adjoining tissues. I want no muscular tissue overlapping the end of the bone. If you have a long muscular flap, it is sure to either slough away or gradually become absorbed. If it should slough, it immediately complicates your case by adding to the dangers from septicæmia."

When he had made his skin flaps he then made a section of the periosteum, extending half way around the shaft of the bone, and six centimeters in length. Having peeled this carefully back, he had a perfect covering for the end of the bone, after the latter was sawn off. He says, that since he has adopted this mode of procedure, he has never had a single case of secondary disease, such as caries or necrosis, following his amputations. When he had sawn off the femur, and brought the skin over the end of the stump, lo! his flaps were so short that they would not come together without considerable tension. He remarked: "As you see, gentlemen, the end of my bone is either too long, or my flaps are too short, and, as I cannot lengthen the latter, I shall have to shorten the former;" and without another word of explanation he proceeded to saw off another six centimeters from the end of his stump.

Before he closed the wound, he applied eighteen ligatures, all of which, except the one to the main branch of the femoral artery, were of carbolized catgut. These ligatures of course were all cut short and left to be absorbed; and I have never seen him leave the long ends of ligatures out of a wound with a view to their future removal. A drainage tube was introduced into the inferior and superior border of the stump, and the whole was sewed together in the most painstaking manner. The stump was dressed with Lister's antiseptic dressing, the whole operation being done under the spray. The operation lasted one hour and ten minutes. In contrast with this time, I have seen Prof. James R. Wood, of New York, do this operation complete in five minutes, making his flaps and sawing off the bone in forty-five seconds.

The result of this operation was most satisfactory indeed. The whole wound healed by first intention, and the patient never had a temperature above 37.5, and never suffered any considerable amount of pain.

In speaking of the rapid recovery of this case, Prof. Langenbeck said that he attributed it mainly to the painstaking manner in which he had arrested *every vestige* of hæmorrhage before closing the wound. He believed that in the vast majority of cases where a constitutional reaction followed surgical operations of this kind, and which we had formerly been taught to look upon as having its origin in an inflammatory reaction, was in fact of a septic character, and that a decomposed blood clot situated at the extremities of open blood vessels, was the very best possible condition for generating such a process.

### PROLAPSUS ANI.

A boy aged six years was brought into the clinic, suffering from this affection. It had troubled him for two years, but until within the last month the gut only came down at the time of the stools. For the last four weeks it had been down constantly, and neither the mother or her family physician had been able to replace it. In connection with the case Prof. Langenbeck said: "In this disease I have been able to cure *every case* in which I have instituted the treatment I shall adopt in this one, where the the patient has been above three years of age.

"Why I have not been equally successful in those of more tender years, I cannot tell, but am disposed to attribute my failures to the fact of younger children being less manageable, and their pressing down efforts when they cry."

His treatment consists in the injection of ergotine into the cellular tissue beside the rectum. He passes the hypodermic syringe parallel with the gut to the depth of about three centimeters, and injects about 20 minims of the solution into each point, usually making the injection at two or three places around the sphincter.

As the mucous membrane of the prolapsed gut in this case was very relaxed and superabundant, he made three parallel incisions through it, about four centimeters in length, with the thermo-cautery, with a view of producing a cicatricial growth that would contract the parts when it healed. This last procedure he did not consider essential to the treatment, but it would probably hasten the cure. The gut was now replaced, and the boy put to bed, and the cure was complete, as the bowel never again came down.

I have been most highly pleased to witness Prof. Langenbeck's operations upon the uterus, and in listening to his lectures upon this subject, not only on account of his wide range of personal experience, but also on account of the fact that I look upon him as a man who is the least influenced by any special hobby of any man that I have ever seen operate on that organ.

### UTERINE POLYPI AND FIBROIDS.

I saw him operate on a woman for the removal of a uterine fibroid polypus that had descended into the vagina and filled this organ so completely that the evacuation of both the rectum and bladder was completely obstructed.

The growth was so large that he was unable to pass the écraseur over the entire mass to its cervical attachment, and hence he had to pass the chain of the instrument around its lower segment, and remove it in three separate portions.

The last was so large that he had to extract it with a pair of obstetrical forceps. He said: "Whether these growths be solid, soft or cystic, there are only three modes of removing them that I recognize as proper to adopt, and these are, the knife or scissors, the écraseur and the galvano-cautery. When they are attached high up in the uterus, I do not like the galvano-cautery, for the reason that you can never be really certain what you are burning, and you may destroy tissues that will lead to disastrous consequences."

The use of the ligature for the removal of these foreign bodies he especially condemned. In the early years of his professional life, he had seen two patients die of septicæmia following the removal of a uterine polypus in this manner.

We must remember, in all our operations upon the uterus, that although we have to do with an organ that will tolerate a good deal of mechanical violence, that we have also to do with a mucous surface that will the most rapidly absorb effete matter of any tissue in the entire body ; and that when a polypus is ligated it soon becomes a putrid mass, from which the whole economy may become rapidly contaminated. The twisting off of polypi he also considers a bad practice, and has seen at least one case where the extensive laceration of the mucous surface of the womb attending the operation resulted in a metritis from which the patient died.

Prof. Schröder had in his wards at the Charité this winter some interesting cases to illustrate his management of different forms of uterine fibroids. In our treatment of these cases, he says, we should remember one fundamental rule, which is, that in the history of every such case there usually comes a time when the morbid growth will cease to increase in size, begin perhaps to undergo a retrograde metamorphosis, and become in time entirely innoxious as far as the well-being of our patient is concerned. Directly opposed to this are the facts connected with the history of most cases of ovarian cysts. Their tendency is ever to increase in size, and their removal will sooner or later be imperatively demanded. Keeping these facts in view, we seize upon the most appropriate time for our operative interference. In the management of a case of uterine fibroid, he says, investigate your case accurately as to this fact. Is its attachment situated upon the lining membrane of the *body* of the womb, or upon the *cervix* uteri. If it have its origin from the body of the womb, you are to consider it as a *noli me tangere*, unless it be the direct cause of symptoms that are likely to prove dangerous to the life of the woman. On the other hand, if it spring from the cervix uteri, you may operate on it in almost any way with comparative safety.

To arrest the hæmorrhages that accompany these cases, he swabs out the inner surface of the womb with either the tincture of iodine or a solution of one of the astringent salts of iron.

A question in which I have been greatly interested, and upon which I have interviewed everybody, is the value of the hypodermic injection of ergotine in the treatment of uterine fibroids.

Prof. Schröder says that he has often seen cases greatly benefited by this treatment, but has never seen a case entirely cured by it. By its use the hæmorrhage will often cease, and the tumor become greatly lessened in size. To test the remedy he says you must use at least one hundred injections. He makes them into the cellular tissue of the abdominal walls, and repeats them as often as every alternate day. As so protracted a use of an agent that is often very painful, taxes to the utmost the patience of both physician and patient, few carry it out thoroughly.

Braun, of Vienna, makes these injections into the outer aspect of the thigh, where they are better borne. He uses also Bourbellon's ergotine, and none other, as he says you never have an abscess follow its use. Its name, I believe, is derived from a Swiss chemist who manufactures it.

Dr. Routh, of Dorset House Hospital for Women and Children, in London, is the greatest enthusiast of any I have met in Europe as regards the efficacy of his treatment of intra-uterine fibroids. His plan is to first puncture the tumor by passing a sharp pointed instrument into it about the size of a number six English catheter. The depth to which he makes this puncture will depend upon the size of the tumor, but will usually be to about one-half of the thickness of the growth. After making his puncture, he introduces into the hole thus made a wire of nearly the same size, heated to a red heat. He claims that in this way you can excite an inflammatory change in the body of the fibroid that will lead to its absorption, and that too without any of the dangers of a septic process following the procedure, which would be likely to occur if you attempted to accomplish the same object in any other way. He says that he has never failed to benefit a case that he has treated in this manner.

He was surprised when I told him that I had seen Prof. Péan, of Paris, carry out the same idea in the management of some cases at the St. Louis.

### AMPUTATION OF THE CERVIX UTERI.

Of the various modes of doing this operation, Prof. Langenbeck prefers the knife or the scissors. The écraseur, he says, should *never* be used for this purpose. He relates a case that occured in his clinic, when Prof. Bilroth was his assistant, in which, although he applied the instrument with great care, and kept his finger on the chain during the whole operation, Prof. B. tightening the screw, a portion of the bladder was included in the incision, and the patient died two days later of peritonitis. The objection that Prof. Langenbeck has to the galvano-cautery in this operation is that you can never be positively sure as to *just* how much tissue you are removing.

On the other hand, since I have been in London I have seen Prof. Barnes amputate the cervix twice with the écraseur. He used the instrument without a speculum, being guided wholly by the index finger of each hand, which he kept in contact with the chain.

After the cervix is removed, he applies the thermo-cautery, to arrest any hæmorrhage that may exist. This, he tells me, is his *uniform* mode of doing this operation, and that he is perfectly satisfied with his results. Prof. Barnes brought out a patient and laid her on the operating table, telling me as he did so, that he was going to show me the American operation (Emmet's).

But on examining the cervix, and seeing it very red and congested, he concluded that he would wait a few days, until the congestion in the parts subsided. In fact, he feared the hæmorrhage that would follow. I could but contrast his timidity with the confidence with which some German operators would have attacked these parts with the knife, without the least fear or hesitation.

I have seen the amputation of the cervix done with scalpel about forty times during my stay in Berlin, and not in a single instance have I seen any serious trouble in arresting the bleeding. Dr. A. Martin, of this city, read a paper last year before the gynæcological section of the German Medical Society, on "The Therapeutics of Chronic Metritis," in which, after going on to state that as all medications, both local and constitutional, had proved of no avail in the management of this disease, he had been induced to resort to an operative procedure for its cure, which operation consisted in an amputation of the cervix uteri.

At that date (August last) he had done the operation 109 times. Of these patients, 72 suffered from a simple chronic metritis, one-half of which number he had had under observation and treatment for many months without the least improvement in their condition. Twenty-six were performed for a cancerous condition of the cervix, and eight for a local hypertrophia of one or both lips of the neck of the womb. Of these cases, only two died as a direct result of the operation. One of these was very anæmic from loss of blood before the operation, and died of septicæmia soon after. The other proved fatal from the supervention of typhoid fever.

His mode of doing this operation is to cut away a portion somewhat funnel-shaped, the base below and the apex above, of from 3 to 5 centimeters in length, depending upon the length of the uterus, as measured by the sound before he operates.

I have followed Dr. Martin carefully for nearly four months, and have seen him and assisted him at this operation more than thirty times; and while I am perfectly sure that no écraseur nor galvano-cautery can do this operation so well as does his knife, I am sorry to be compelled to believe that, like some other of our noted gynæcologists, he is bent on doing "*his*" operation as often as possible, and so submits many a poor woman to this treatment, who might be relieved by a more simple procedure.

Unfortunately, like many another celebrated operator, he is inclined, I fear, to keep in the background as much as possible the accidents that follow his treatment.

I saw a woman in the service of another gynæcologist of Berlin, operated on for complete occlusion of the os uteri and hæmatometra, that followed an amputation of the cervix by Dr. Martin.

When I related the case to Dr. M., he said that this patient was one of two who had had secondary hæmorrhage following the operation, and he attributed this bad result in her case to the extra stitches that he had had to put in, in order to stop the bleeding, as well as her having left his service and passed out from under his observation sooner than she should have done. Prof. Schröder's mode of amputating the cervix, and which Prof. Langenbeck recommends, is, in my mind, at once the most simple and easy of execution of any of the numerous modes of doing this operation that I have seen tried. Dr. Martin, however, objects to it because the resulting stump is somewhat flat, while when done after his plan (Dr. Martin's) the stump is conical in shape. Prof. Schröder's reply to this is, that nature has to re-shape the parts after any cutting procedure of this kind, and that in the end his operation leaves as natural a cervix as any other. To do this operation Prof. Schröder places his patient on her back, her thighs flexed upon the abdomen and the legs upon the thighs. (I have never seen a German operator place a woman in Sims' position).

A Simon's speculum is next introduced, and the perineum pressed well down. A Hegar's irrigator containing a 5 per cent. solution of carbolic acid is now made to throw a constant stream of this fluid upon the cervix uteri during the entire operation. In fact, Prof. Schröder claims to make this operation strictly antiseptically, substituting the fluid carbolized solution for the spray. A pair of scissors are now introduced into the os uteri, and the os incised as high up as the vaginal insertion on each side. The cervix is thus divided into two flaps, and while an assistant pulls down upon and steadies the womb by means of a pair of forceps made fast to one flap, the surgeon seizes the other and amputates it rapidly with the knife, cutting, of course, from below upward, so that you have left after the amputation a somewhat long flap-shaped mucous membrane to cover the stump.

By means of a curved needle, armed with a heavy silk ligature, two sutures are now passed from the vaginal mucous surface of the stump into the os. These sutures are each placed 1 centimeter from the median line at their point of entry into the vaginal walls. They are now tied tightly, and serve to render the os uteri patulous, as well as to pull down and hold the womb firmly, while the other lip is being cut away in the same manner. From three to four stitches are next applied on each side of these central ones, and the operation is completed. The vagina is now tamponed well with salicylated cotton, which seals up the wound and makes the whole procedure antiseptic.

With the uterus thus firmly held, and with a needle curved to a semi-circle and armed with a silk thread, there is but little difficulty in passing a suture through the muscular walls of the uterine stump in such a manner that when it is tightly tied it will include any bleeding vessel and effectually arrest any hæmorrhage.

German operators do not use the silver wire for sutures, either for this operation or Emmet's, as they claim that the silk answers every purpose, and is much easier to apply and remove.

Prof. Langenbeck lays down one very emphatic rule as to the use of styptics to arrest hæmorrhage where operations have been done in the vagina, and where the probabilities are that the peritoneum has been encroached upon. *Never*, he says, use preparations of *iron* for this purpose. He relates several cases where he used a solution of iron, and after his patients died of peritonitis he found that the walls of the peritoneum had sloughed away from the effect of his styptic. He believes that even a moderately strong solution of iron will cause a necrosis of any serous membrane to which it is applied.

Prof. Freund, late of Breslau, but now called to Strasburg to fill the place of Prof. Gusserow, appointed as Professor of Midwifery in the University of Berlin, spent a month in the latter city this winter. As the reader perhaps knows, Prof. Freund is the father of the revised method of extirpation of the entire uterus through the abdominal walls.

I saw him operate on a case for Dr. Schröder just before I left Berlin. The patient was alive four days after the operation, but her recovery was considered doubtful. I saw him begin another operation of the same kind for Dr. Martin, but after making his abdominal incision, he found the pelvic glands so invaded by cancerous infiltration that he would not attempt the removal of the uterus.

He closed up the wound, and Dr. Martin then performed his amputation of the cervix uteri as a palliatory expedient. This poor woman had a good deal of constitutional disturbance, but was convalescing the last time I saw her, ten days after the operation.

Prof. Freund has done this operation of the total extirpation of the womb 12 times and has had 7 recoveries, while in Dr. West's tabulated 25 cases there occurred 23 deaths.

The enucleation of the womb entire through the vaginal walls, of which a lately published case is reported by Prof. Lane (I believe) of San Francisco, Prof. Langenbeck considers an anatomical *impossibility*.

He says, that while you may with considerable facility separate the *cervical* portion of the womb from its attachment, that when you reach the *body* of that organ, you cannot separate its muscular tissues from its peritoneal coating without the greatest difficulty, even when you have the uterus removed from the body and dissect with the greatest care, and as an operative procedure upon the living body he does not think its accomplishment possible.

### TRACHEOTOMY.

A child six years old was brought into Prof. Langenbeck's clinic, and being placed upon the table. He said :

" Gentlemen, you would scarcely imagine on looking at this little boy and seeing how little his respiration is embarrassed, that I am about to open his trachea."

The patient was suffering from dipththeria, and had twenty-four hours previously developed a croupy cough. Prof. L. laid it down as a rule, to which there should be no exception, that in the management of these cases that you should operate before signs of extreme dyspnœa and blood poisoning come on. Waiting will do no good, for with an almost absolute certainty tracheotomy will be necessary, and the longer you wait the less are your chances of success. The operation itself is not dangerous unless done when the patient is in extremis. Since 1870 he has done this operation 700 times in this clinic. In some years he has saved as high as 40 per cent. of his cases operated on, and in others only 10 per cent.

He was not able to explain why there should be this great difference in the mortality of his cases in different years; but one fact he did know, and that was that since he had given out word to the physicians of Berlin that they should send their diphtheritic patients earlier for this operation, that his success had been much better.

In the City hospital of Berlin diphtheritic patients are kept in a ward adjoining that devoted to the chronic diseases of women. On making one of my evening visits to this institution with Dr. Alberts, physician in charge, he showed me a boy ten years old who had just been brought in with this disease, and upon whom he proposed to perform the operation of tracheotomy if the case did not improve after one hour.

I administered the chloroform, and the doctor cut down through the skin and underlying fascia, and with great care separated, as he thought, the two lobes of the thyroid gland with the handle of his scalpel, and finally reaching what he thought to be the trachea, made an incision into it. No air escaped, though he pulled the edges of the wound open with a pair of forceps. The true condition of things now flashed over him in an instant. He had gone outside of and behind the trachea, and had opened into the œsophagus. After waiting a few moments and expressing his great horror at what he termed his *extreme stupidity*, he opened the trachea and put in the tube. With even this mishap this case did well as far as the operation was concerned, though the patient died five days later from extension of the diphtheritic process into the bronchi. The boy was not given any nourishment by the mouth for 24 hours, but after that time he was given fluid nourishment, which passed into the stomach without hindrance.

W. S. CALDWELL.

LONDON, March 3d, 1879.

